# Evolución y efectividad de los sistemas adhesivos de séptima y octava generación en restauraciones directas. una revisión

**DOI:** 10.21142/2523-2754-1104-2023-178

**Published:** 2023-12-28

**Authors:** Kemberly Bredgette Chacón Gahona, Byron Roberto Morales Bravo, Santiago Efraín Vintimilla Coronel, Patricio Fernando Sarmiento Criollo

**Affiliations:** 1 Carrera de Odontología, Universidad Católica de Cuenca. Cuenca, Ecuador. kemberly.chacon@est.ucacue.edu.ec, bmorales@ucacue.edu.ec, svintimilla@ucacue.edu.ec, psarmiento@ucacue.edu.ec Universidad Católica de Cuenca Carrera de Odontología Universidad Católica de Cuenca Cuenca Ecuador kemberly.chacon@est.ucacue.edu.ec bmorales@ucacue.edu.ec svintimilla@ucacue.edu.ec psarmiento@ucacue.edu.ec

**Keywords:** sistemas adhesivos, generaciones de sistemas adhesivos, evolución, restauraciones directas, adhesive systems, generations of adhesive systems, evolution, direct restorations

## Abstract

**Introducción:**

La adhesión dental es la encargada de los procesos químicos de unión dentino-esmalte. Para una correcta elección, previa a su aplicación en tratamientos restauradores, es importante conocer la generación a la que pertenece, sus propiedades, técnicas de aplicación, etc., con el fin de garantizar el éxito en los procedimientos. Actualmente, los sistemas adhesivos han evolucionado y se presentan en el mercado 8 generaciones, cada una con un mejor estándar de calidad que la anterior.

**Objetivo:**

Identificar qué generación de sistema adhesivo es la más viable para ser aplicada en tratamientos de restauraciones directas de piezas dentales.

**Materiales y métodos:**

Se revisó y recopiló 133 artículos publicados en los últimos 20 años. Tras analizarlos, de acuerdo con su relevancia clínica, se excluyeron 88. Los artículos restantes fueron reanalizados y se seleccionaron únicamente aquellos enfocados en describir las generaciones de sistemas adhesivos aplicadas en restauraciones directas, con lo que quedó un total de 56 artículos, que incluyeron reportes de casos clínicos. Se utilizó el buscador Google Académico, SciELO y bases de datos de interés, como PubMed, Scopus y Medigraphic. Tres revisores realizaron independientemente el análisis y búsqueda de datos, y se emplearon las siguientes palabras clave: sistemas adhesivos, generaciones de sistemas adhesivos, evolución, y restauraciones directas.

**Resultados:**

Se evaluaron 56 artículos, según su relación con el tema y la información previamente buscada. Se determinó que la séptima generación de sistemas adhesivos es la más viable para ser aplicada en tratamientos dentales, debido a las mejoras que presenta en sus características y a los resultados con buen pronóstico, mencionados tanto por profesionales odontológicos como en diversas investigaciones.

**Conclusión:**

El avance de la tecnología implica nuevos conocimientos, por ende, la efectividad de los sistemas adhesivos ha cambiado notablemente. La evidencia científica ha demostrado que los adhesivos de séptima generación presentan una mayor efectividad en las restauraciones directas.

## Abstract (en)

**Introduction:**

: Dental adhesion is responsible for the chemical processes of dentin-enamel union for a correct choice before its application in restorative treatments; it is important to know properly the generation to which it belongs, its properties, application techniques, etc. All this is to ensure success in the procedures to be performed. Currently, adhesive systems have evolved, and 8 generations are available in the market, each with a better-quality standard than the previous one.

**Objective:**

To identify which generation of the adhesive system is the most viable to be applied in direct dental restorative treatments.

**Materials and methods:**

We reviewed and compiled 133 articles published from 20 years ago to date. After analyzing them according to their clinical relevance, 88 were excluded, the remaining articles were reanalyzed, of which only those focused on describing the generations of adhesive systems applied in direct restorations were selected, leaving a total of 56 articles, which included clinical case reports. The Google Scholar search engine, SciELO and databases of interest, such as PubMed, Scopus and Medigraphic, were used, three reviewers independently performed the analysis and search of data, and the keywords were used; adhesive systems, generations of adhesive systems, evolution, and direct restorations.

**Results:**

: 56 articles were evaluated, according to their relationship with the subject and with the information previously sought, it was determined that the seventh generation of adhesive systems is the most viable to be applied in dental treatments, due to the improvements it presents in its characteristics, and the results with good prognosis mentioned by both dental professionals, as in research.

**Conclusion:**

The advancement of technology implies new knowledge; therefore, the effectiveness of adhesive systems has changed significantly. Scientific evidence has shown that seventh-generation adhesives are more effective in direct restorations.

## INTRODUCCIÓN

En la odontología restauradora, los adhesivos se encargan principalmente de la unión dentino-esmalte. Para ello, es importante determinar la naturaleza, la fuerza adecuada de adhesión, las propiedades, las técnicas de aplicación, las nuevas generaciones, etc. Conforme han pasado los años, estos sistemas han sufrido cambios evidentes, de manera que han revolucionado su uso en la práctica odontológica. Es importante garantizar una excelente adhesión, puesto que la resistencia final de las restauraciones dependerá totalmente de este procedimiento. El principio de esta unión es crear una capa híbrida por medio de la interpenetración de monómeros en el tejido duro de la pieza dental. Cuando la resina logra la polimerización, se crea un enlace estructural idéntico al vínculo antes mencionado entre la dentina y el esmalte ^1^.

El desarrollo de adhesivos dentales que cuenten con mejores características y, a la vez, sean más fáciles de manejar se origina ante la necesidad de reducir el tiempo de trabajo o proceso clínico de los tratamientos. Desde hace ya varias décadas, los dentistas se han encontrado con materiales químicos que, para su uso, tenían que cumplir una secuencia de pasos, y al mismo tiempo mezclarse en un orden específico con el objetivo de lograr una correcta unión micromecánica de la restauración y el diente ^2^.

Siempre ha estado presente la inquietud de descubrir el material restaurador ideal para realizar tratamientos dentales. Entre las características que debe tener, se considera que sea realmente adhesivo a la estructura del diente ^3^. Son muchos los principios que juegan un papel importante al garantizar una adhesión a la estructura dental; es verdaderamente difícil analizar los factores y, sobre todo, comprender cada principio de adhesión con la dentina. Aún no se ha establecido una clasificación íntegra de los sistemas adhesivos que sea capaz de agrupar a todos y cada uno de estos de manera eficiente. En el pasado y presente, algunos autores clasificaban estos sistemas adhesivos según generaciones, y cada vez que se lanzaba al mercado un nuevo material era propuesta una nueva generación. Esta clasificación fue considerada errónea y confusa, debido a la idea incorrecta de que mientras más reciente era la generación mejores propiedades adhesivas tendría. En la actualidad, para garantizar un buen sistema adhesivo, se tienen en cuenta varias características, entre ellas su forma de polimerizar, según afinidad a fluidos dentinarios, según la cantidad de pasos que necesite, etc. Esta alternativa incluye a todos los sistemas, independientemente de su tiempo de fabricación o generación ^4^.

Hoy existen diversas técnicas que mejoran la integridad marginal en las restauraciones, cuidan el área dentino-pulpar, conservan la restauración a largo plazo, facilitan la técnica y reducen el tiempo de trabajo clínico. Para conseguir una buena adhesión a las estructuras dentarias, se pueden utilizar sistemas adhesivos que actúen como agentes acondicionantes, por ejemplo, los autograbantes o de 7.^a^ generación. Otra opción a tener en cuenta son los adhesivos de 8.^a^ generación, que presentan en su estructura generaciones previamente elaboradas y reconfiguradas únicamente con propósitos de mercado ^5^^,^^2^.

El objetivo de esta revisión fue describir y revisar cada sistema adhesivo con el fin de brindar la información necesaria acerca de la evolución de los sistemas adhesivos, comparar los adhesivos de 7.^a^ y 8.^a^ generación con sistemas anteriores, y determinar cuál de todos es el más viable para restauraciones directas, lo que permitirá al odontólogo realizar una adecuada selección y uso del sistema, todo esto mediante una revisión actualizada.

## MATERIALES Y MÉTODOS

Se efectuó una búsqueda en bases de datos digitales (Researchgate, ScienceDirect, PubMed, Elsevier, Dialnet Plus, Medline), mediante las palabras claves MeSH “Sistemas adhesivos”, “Generaciones de sistemas adhesivos”, “Técnicas”, “Evolución”, “Dentina”, “Esmalte”, “Protocolo” y “Restauraciones directas”. Además, se buscaron artículos en relación con el tema en revistas de alto impacto, como *Revista de la Facultad de Odontología, Revista de Operatoria Dental y Biomateriales, Oper Dent, Revista Cubana de Estomatología, Dent Mater, Revista ADM, Journal of Dental Research, RCOE, Revista Facultad Odontología UBA, Revista Dental de Chile, International Journal of Odontostomatology.*

Entre los criterios de inclusión se consideró estudios de reportes de casos, revisiones de la literatura, estudios retrospectivos, descriptivos y observacionales. Se tomó en cuenta artículos de los últimos 20 años, sin restricción de idioma y de acceso libre. En cuanto a los criterios de exclusión, no se tomaron en cuenta monografías, cartas al editor ni artículos de opinión. Se analizó el resumen y el título, y finalmente se seleccionó un total de 56 artículos de acuerdo con los parámetros de la investigación.

### Sistemas de adhesión

Al hablar de las mejoras que han surgido a través del tiempo en la odontología restauradora, los sistemas adhesivos son un tema con alto impacto, puesto que su constante evolución cambió totalmente la práctica odontológica. Estos sistemas modificaron diversas teorías con respecto a preparaciones cavitarias innecesarias y preservación de estructura dentaria sana, lo cual se considera como lo más relevante del tema ^6^.

Actualmente, en el área comercial odontológica se encuentran sistemas adhesivos cada vez más confiables, con mejores componentes, mayor facilidad de manejo y menos pasos para su aplicación, todo esto con el fin de reducir el trabajo clínico, evitar errores en su utilización y obtener mejores resultados en los tratamientos restauradores ^7^.

Su desarrollo ha tenido como objetivo el uso de sistemas menos complejos, por lo que surge un sistema de autograbado y se evita el uso del conocido grabado ácido total ^8^.

La adhesión es un proceso vital ya que es necesaria para soportar la fuerza de contracción producida en la polimerización de la resina, proporcionar integridad marginal y una correcta retención de la pieza dental restaurada ^9^. Además, incluye pasos imprescindibles para establecer la respectiva unión entre el material de resina y el esmalte o dentina, según el caso ^10^.

Los sistemas adhesivos se consideran uno de los principales conjuntos de materiales en la odontología restauradora y estética ^11^. Se presentan como una solución de monómero de resina y están compuestos por agrupaciones con propiedades hidrófilas e hidrófobas; asimismo, contienen solventes, cargas inorgánicas, estabilizadores, etc. Pese a estas características, es preciso tener en cuenta la anatomía dental de la pieza que se va a tratar y realizar un examen completo de las estructuras, como esmalte, dentina y tejidos de soporte, para evitar complicaciones e interpretar como se relacionan los enlaces adhesivos con el tema ^12^. Estos biomateriales actúan de manera tópica, ocluyendo los túbulos dentinarios y bloqueando mecánicamente estímulos extrínsecos, la exposición tubular que se produce en el proceso se considerara inerte durante la preparación cavitaria ^13^.

### Evolución de la odontología adhesiva

Los adhesivos en la odontología restauradora tienen una larga e innovadora historia. Su aplicación en los trabajos clínicos, el éxito o fracaso del tratamiento ha sido justificado, conforme el tiempo ha transcurrido, con su aplicación en estudios e investigaciones científicas. Buoncore, el pionero de la adhesión, le dio inicio a través de la obtención de adherencia en resina acrílica, mediante el uso de ácido fosfórico; desde entonces, el objetivo de la práctica dental se ha enfocado en generar vínculos estables y eficientes entre los componentes. Uno de los primeros materiales adhesivos fue el Sevriton Cavity Seal, creado en 1949 por Oskar Hagger, el mismo se introducía en la superficie dentinaria ^14^.

La segunda generación fue creada en 1970, entre sus componentes se encontraba BisGMA y HEMA ^15^.

Años más tarde, Nakbayashi *et al*. (1982) introdujeron el concepto de capa híbrida y descubrieron que su formación se producía luego que la dentina fuera tratada con ácido y enjuagada con agua. Esta capa era hidrofóbica, resistía ácidos y contenía colágeno, cuando esta superficie se humedecía con adhesivo, se transformaba en un componente hibrido para la dentina y la resina. ^16^. Más adelante se relacionó la tensión provocada por la contracción con resina compuesta polimerizada a través de químicos y luz ^17^.

Posteriormente, en 1988, Fusayama aseguró que los compuestos químicos aplicados en la dentina garantizaban una mejor estabilidad de retención en cuanto a la vitalidad pulpar ^18^.

Un poco más tarde, la adhesión tuvo un significante aporte planteado por Kanca, autor que introdujo la “adhesión húmeda”, y manifestó la importancia de que la dentina se conserve húmeda posteriormente al grabado, con esto se obtendrían mejores resultados en cuanto a la fuerza de unión ^19^.

Dicho descubrimiento marcó un gran cambio en la adhesión dental, debido a que el secado de la dentina modifica la red de colágenos, lo que disminuye significativamente su volumen y crea una superficie mucho más impermeable; esto evitaría la polimerización y penetración de monómeros en la dentina desmineralizada. Se determinó, además, que la humedad adecuada presente en el procedimiento garantizará el éxito de esta técnica. En otras palabras, la adhesión se desarrolla mediante el remplazo de minerales dentales por monómeros resinosos que se entrelazan mecánicamente en las porosidades de la pieza dental ^20^.

### Clasificación de los sistemas de adhesión dental

Con el paso del tiempo los sistemas adhesivos se han clasificado de diversas maneras, de acuerdo con su generación, el número de pasos que implique su aplicación, el método para realizar el grabado, el número de envases que se utilicen en el tratamiento clínico, etc. ^21^.

En su clasificación, encontramos una gran variedad, entre ellos los siguientes:

a. De acuerdo con su eliminación o modificación del barrillo dentinario

b. De acuerdo con el agente grabador

- Grabado y lavado

- Autograbado

c. De acuerdo con el sistema de activadores que posee:

- Auto o químico-polimerizables

- Fotopolimerizables

- Duales

d. De acuerdo con su evolución histórica

- 1.a generación 

- 2.a generación

- 3.a generación

- 4.a generación

- 5.a generación

- 6.a generación

- 7.a generación

- 8.a generación ^22^

### Generaciones de sistemas de adhesión dental

Como ya se mencionó, la odontología restauradora ha sido testigo de un sinnúmero de avances con el pasar de los años, desde la aplicación de amalgamas como parte de tratamientos dentales, hasta el universo de resinas que se encuentran presentes actualmente en el mercado. La adhesión y sus respectivos sistemas también se consideran un tema vital en el área odontológica, ya que se evidencian 8 generaciones de adhesivos ^23^.

Primera generación. Uno de los objetivos de esta generación se basó en obtener una correcta compatibilidad de la cavidad bucal con el adhesivo. Su poca capacidad de adhesión provocaba filtraciones al momento de utilizarla, todo esto a causa de la hidrólisis que ocurría frente a la presencia de saliva y adhesivo ^(24, 25)^. Pese a que su aparición a finales del año setenta no fue considerada como algo novedoso, entre sus características recalcó su gran fuerza de adhesión al esmalte; de igual manera, su baja adhesión a la dentina fue otro punto evidente de esta generación. El proceso de adhesión se conseguía a partir de la quelación del adhesivo con el calcio presente en la dentina, esto provocaba posteriormente desprendimientos ^26^. Entre las recomendaciones del fabricante se encontraba su aplicación para cavidades de clase III y clase V ^27^.

Segunda generación. En esta generación se evidenció una mejora notable en cuanto a la unión y solidez que se presentaba en el esmalte y la dentina a la hora de utilizarla en procedimientos dentales. La adhesión de esta generación se fundamentaba en una reacción de tipo fosfato-calcio, en la que era indispensable el uso de resinas de dimetacrilato, puesto que no se recomendaba el uso de la reconocida resina Bis-GMA ^28^. Su desarrollo ocurrió a inicios de los 80 y se utilizó como parte del sustrato adhesivo al barrillo dentinario ^(29, 30)^. Presentaba una baja capacidad de adhesión por lo que la preparación cavitaria aún era necesaria; además, se evidenció una alta sensibilidad posoperatoria y posible microfiltración en la cara oclusal de restauraciones posteriores; esto cuestionó significativamente su uso en los tratamientos dentales ^31^^,^^32^.

Tercera generación. Esta generación se presentó a finales de los ochenta y se consideró como la pionera en el uso de primer y adhesivo, fue vital la colocación del agente o ácido, ya que este mejoraba la permeabilidad y adhesión en la dentina, redujo la necesidad de realizar una preparación cavitaria para mejorar la retención, y la sensibilidad posoperatoria disminuyó considerablemente. Entre sus desventajas, la longevidad fue la que más resaltó, puesto que 3 años después de su colocación en boca esta perdía los efectos de retención adhesiva; pese a esto, el uso de dos componentes en un solo sistema adhesivo dio inicio a una odontología mucho más conservadora, hasta la actualidad muchos profesionales continúan utilizando estos sistemas ^28^^,^^33^^,^^34^.

Cuarta generación. En esta generación ya se encuentra la técnica de grabado total y la eliminación del barrillo dentinario, mediante el ácido ortofosfórico se graban simultáneamente el esmalte y la dentina, su aparición se dio a inicios de los noventa, revolucionó la odontología en diferentes aspectos. De igual manera, tuvo gran acogida ya que mejoró la fuerza de adhesión y disminuyó la sensibilidad posoperatoria; además, sustituyó por resina a la hidroxiapatita y el agua de la superficie dentinaria, lo que perfecciona la adhesión ^28^^,^^35^^-^^37^.

Quinta generación. Con el fin de obtener una adhesión química y reducir pasos, la quinta generación busca conformar una técnica con mejor adhesión y menor sensibilidad. La mayoría de estos sistemas incorporaron en su técnica el acondicionamiento de la dentina y el esmalte. Es considerada una de las mejores generaciones debido a su fácil adhesión tanto en la dentina o el esmalte de la pieza dental, como en cerámica y metal, y por su presentación todo en uno, es decir, en un solo frasco, el componente del frasco no necesita mezcla, lo que implica un menor índice de error, y es idóneo para cualquier tratamiento dental ^28^^,^^38^.

Sexta generación. Esta generación se encarga de omitir el grabado mediante ácido e incorporan en su técnica imprimador de tipo autograbable mezclado con adhesivo e imprimador. Entre los principales componentes de esta generación se encuentra un líquido acondicionador de dentina, el proceso ácido en la dentina se produce de manera autolimitada, y el derivado del grabado se integra a la interface dental restaurativa de manera permanente. Cabe recalcar que, a pesar de esta situación, la adhesión dentinaria se conserva en boca y se obtienen buenos resultados; sin embargo, en el esmalte se exponen dudas acerca de la adhesión sin previo grabado y preparación ^28^^,^^39^^,^^40^^).^

Séptima generación. Esta generación se la conoce con el nombre de todo en uno, se caracteriza por ser autograbante y en el mercado se lo encuentra en un solo frasco. Simplifica, además, los diversos materiales presentes en la sexta generación y usa un solo componente. Es apta para autograbado, adhesión autocondicionante, y no provoca sensibilidad posoperatoria ^39^^,^^41^.

Octava generación. Posee en su estructura un relleno nanométrico de monómero hidrófilo ácido, es útil en el manejo de restauraciones directas e indirectas, apto tanto en dentina como en esmalte pese a la presencia de contaminación con humedad o fluidos, y está disponible en autograbado o grabado total ^42^^,^^43^^).^

**Tabla 1:** Fuerza MPa de las diversas generaciones de sistemas adhesivos ^44^

Generación de agente adhesivo	Fuerza de MPa
Primera generación	1-2 MPa
Segunda generación	2-8 MPa
Tercera generación	8-15 MPa
Cuarta generación	17-25 MPa
Quinta generación	20-25 MPa
Sexta generación	18-23 MPa
Séptima generación	18-35 MPa

### Ventajas y desventajas en los sistemas adhesivos de generaciones pasadas y actuales

Adhesivos de tres pasos. Necesitan grabado ácido, tanto en esmalte o dentina, además de un lavado y secado, agente imprimador y adhesivo. Previo al empleo de la resina, se produce la desmineralización de tejidos, la superficie dental que es hidrofílica se convierte en hidrofóbica mediante el primer; de este modo se une la resina adhesiva.

Entre sus componentes se encuentran monómeros polimerizables hidrofílicos diluidos en agua, etanol o acetona cuya función principal es transportar monómero por medio del tejido previamente grabado ^44^. Desplazan los residuos de agua y mejoran la inserción de los monómeros polimerizables en los túbulos dentinarios, y obtiene como resultados tejidos totalmente infiltrados, con la condición de que estos contengan la suficiente humedad en su estructura. Entre las desventajas más notables de estos sistemas, se encuentra la gran capacidad que tienen para adquirir una buena resistencia de adhesión en el esmalte y dentina, existe una alta sensibilidad causada por los diversos pasos que se deben ejecutar para su aplicación, y existe el riesgo de humedecer o resecar la dentina cuando se realiza el lavado y secado posaplicación del agente grabador o ácido ^45^^,^^46^.

Adhesivos de dos pasos. No existe mucha diferencia entre este sistema y el anteriormente mencionado de tres pasos, excepto por su sensibilidad a la técnica, la misma que es mayor que la de su precursor, no se realiza la imprimación de manera independiente. Es vital la aplicación de la técnica de adhesión húmeda, y en los tejidos de igual manera se encuentran húmedos; esto evita el proceso de infiltración incompleta del sistema adhesivo. Es complejo obtener un correcto grado de humedad, entre sus ventajas se encuentra la simplificación de la técnica, la eliminación de la fase de lavado y la reducción del tiempo de trabajo.

Adhesivos de un solo paso. Estos incorporan el grabado ácido, adhesión e imprimación en un solo paso, entre sus ventajas se presenta su fácil aplicación, eliminación del lavado de superficie, únicamente precisa su secado previo a la fotopolimerización ^47^. La técnica de aplicación se ha reducido extensamente manteniendo en un solo frasco todos los componentes necesarios para que se pueda dar la desmineralización dentinaria y el funcionamiento del sistema adhesivo ^48^.

### Protocolo de aplicación de los sistemas de adhesión universales

Este sistema de adhesión puede aplicarse siguiendo 3 protocolos diferentes entre cada uno:

a. Técnica de grabado total

• Desinfectar y lavar la preparación. 

• Aplicar ácido fosfórico en concentración del 37% sobre esmalte durante 15 segundos, y en dentina por 10 segundos. 

• Lavar con abundante agua la preparación, secar con torunda de algodón o papel absorbente, evitar resecar la dentina. 

• Aplicar una sola capa del adhesivo universal de elección sobre esmalte y dentina, frotar durante 20 segundos únicamente en dentina.

• Fotopolimerizar la cavidad dental durante 20 segundos. 

• Realizar la restauración de la pieza dental.

b. Técnica de grabado selectivo

• Desinfectar y lavar la preparación. 

• Aplicar ácido fosfórico en concentración del 37% en el borde cavo superficial del esmalte, y sin abarcar la dentina, durante 15 segundos.

• Lavar con abundante agua la preparación, secar con torunda de algodón o papel absorbente, evitar resecar la dentina. 

• Aplicar una sola capa del adhesivo universal de elección sobre esmalte y dentina, frotar durante 20 segundos únicamente en dentina.

• Fotopolimerizar la cavidad dental durante 20 segundos. 

• Realizar la restauración de la pieza dental.

c. Técnica con adhesivo universal autograbante

• Desinfectar, lavar y secar la preparación.

• Aplicar el adhesivo universal de elección sobre esmalte y dentina no grabada, frotar en dentina durante 20 segundos, volatilizar el adhesivo de manera no direccional con aire a través de le jeringa triple.

• Fotopolimerizar durante 20 segundos.

• Realizar la restauración de la pieza dental ^48^.

## RESULTADOS 

**Figura 1:**
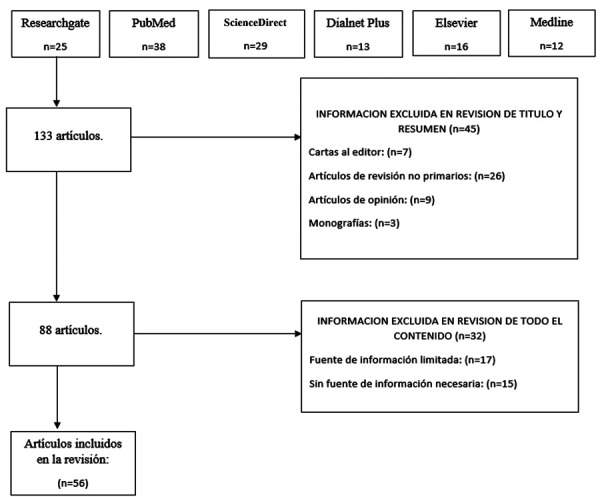
Diagrama de flujo para la selección de artículos (Elaboración propia)

Inicialmente, se recopilaron 133 estudios mediante búsquedas en bases de datos. El diagrama de flujos que se muestra en la Figura 1 incluyó 25 estudios recolectados de Researchgate, 38 de PubMed, 29 de ScienceDirect, 13 de Dialnet Plus, 6 de Elsevier y 12 estudios de la base de datos de Medline. Del total de 133 artículos identificados en la búsqueda bibliográfica, se excluyeron 45 (cartas al editor: 7, artículos de revisión no primarios: 26, artículos de opinión: 9, monografías: 63) y quedaron un total de 88 artículos, los cuales fueron nuevamente analizados de acuerdo con los criterios de inclusión y se excluyeron 32 (fuente de información limitada: 17, sin fuente de información necesaria: 15), Finalmente, 56 artículos fueron incluidos en esta revisión bibliográfica. La selección de artículos y búsqueda bibliográfica se representa en la Figura 1.

## DISCUSIÓN

La odontología restauradora ha sido testigo de múltiples avances y los cambios producidos en esta área se enfocan cada vez más en mejorar componentes, simplificar técnicas clínicas, evolución de materiales, etc. Múltiples estudios e investigaciones mencionan el evidente interés por parte del profesional odontológico en sistemas adhesivos que cuenten con resultados óptimos para los procedimientos dentales, una opción a tener en cuenta son los sistemas adhesivos de séptima generación que presentan una ligera desmineralización en esmalte, aumentan la resistencia de unión y fuerza, reducen la sensibilidad posoperatoria, protocolo y técnica. Otras características importantes a tener en cuenta son la resistencia a fuerzas tensoras traccionales y compresoras, su facilidad de manipulación, y compatibilidad con tejidos dentales, etc. Gracias a estos factores, la séptima generación de sistemas adhesivos ha sido bien acogida por parte del personal odontológico, por lo que es considerada óptima al ser aplicada en tratamientos dentales.

Varios autores coinciden en que es necesario evaluar cada una de las diversas generaciones de sistemas adhesivos presentes en el mercado. En la actualidad, para que el profesional seleccione una de estas opciones, se deben tener en cuenta ciertos aspectos críticos y vitales, con el propósito de presentar tratamientos seguros, confiables, con buena estética y que cumplan cada una de las expectativas del paciente, en el menor tiempo posible.

La adhesión en la odontología busca constantemente componentes cuyas propiedades estéticas y funcionales sean compatibles con las biológicas, puesto que estos pueden encargarse de la función del tejido en el órgano natural, lo que simula las características propias del mismo en su respectivo entorno biológico ^49^.

Es necesario mencionar que actualmente se busca realizar procedimientos ligeramente invasivos mediante restauraciones que requieran una adhesión con escasa densidad, es decir, sean mínimamente finas, y se obtenga como resultado una técnica reducida, buena estética y que eviten desgastar estructuras dentales sanas ^50^.

Buonocore ^51^ menciona que la adhesión dental es un proceso exitoso y de larga duración, pero que en dentina se vuelve dificultoso, ya que tiene estructura orgánica en sus componentes. Zecin-Deren *et al*. ^52^ concuerdan en que una buena adhesión no solo debe relacionarse con un sistema adhesivo, sino también con sus componentes. También resaltan que el esmalte, gracias a su estructura, se considera el tejido dental óptimo para el proceso de adhesión, y para evitar errores en este sustrato resulta vital que tanto el ácido del sistema adhesivo como la resina dispongan de suficiente humedad.

Al contrario, Villa y Morada ^53^ concluyeron que la fuerza de la superficie de la dentina en la unión adhesiva se incrementaba cuando esta era grabada con ácido fosfórico. De la misma manera, Lozada y Rayo ^54^ consideraban a aquel grabado como invasivo si se realizaba en la dentina.

En el estudio realizado por Loguercio y Reis ^55^ se tomó como referencia una comparación de estructuras fisiológicas de la dentina y esmalte, y se concluyó que el ahorro de tiempo en odontología no se considera la mejor opción, ya que diversas casas comerciales, debido al interés financiero, no siempre presentan un buen producto, con bases científicas; por el contrario, únicamente poseen objetivos lucrativos. Al tomar en cuenta esto, el resultado del estudio fue ideal para el sistema adhesivo convencional. Por su parte, también se indicó en que en adhesivos convencionales y acondicionantes, se crea una capa híbrida, la cual, entre sus propiedades posee menos calibre cuando se aplica en sistemas adhesivos autocondicionantes ^54^. 

Por su parte, Garrofe *et al*. ^43^ indican la importancia del seguimiento total de las indicaciones de uso o aplicación que las casas comerciales presentan, pues al respetar estas consideraciones se obtendrá un mejor resultado por parte del adhesivo, y este cumplirá cada una de sus funciones. Además, comentaron que al realizarse un secado excesivo se produciría un colapso de las fibras colágenas presentes en la estructura dental, lo que generaría inconvenientes al impregnarse en el adhesivo.

### Limitaciones

Aun cuando este trabajo de investigación seleccionó los artículos más destacados en la literatura científica sobre este tópico de estudio, una generalización completa no se puede hacer, y estudios que midan la calidad de la evidencia quedándose solo con la más alta evidencia deben ser realizados.

## CONCLUSIONES

Con los sistemas de adhesión universales de séptima generación se pueden obtener buenos resultados en cuanto a la adhesión dentinaria y de esmalte. Se debe tener en cuenta la importancia de su correcta elección de acuerdo con las necesidades de cada tratamiento, la sensibilidad posoperatoria es un factor a tener presente. Estos sistemas presentan resultados satisfactorios en cuanto a este punto, su fórmula es mucho más actual que la de generaciones pasadas. La imprimación y adhesión de la superficie dental se dan de manera simultánea, no es sensible a la humedad residual en la superficie de la preparación, y la fuerza de adhesión en dentina y esmalte es la misma; se consideran biocompatibles, presentan versatilidad y constan de respaldos científicos con buenos pronósticos.

## Fuente de financiamiento:

Autofinanciado
